# Challenges and Opportunities for Urban Environmental Health and Sustainability: the HEALTHY-POLIS initiative

**DOI:** 10.1186/s12940-016-0096-1

**Published:** 2016-03-08

**Authors:** Sotiris Vardoulakis, Keith Dear, Paul Wilkinson

**Affiliations:** Environmental Change Department, Centre for Radiation, Chemical and Environmental Hazards, Public Health England, Chilton, OX11 0RQ UK; Duke Global Health Institute, Duke Kunshan University, Kunshan, 215316 China; Department of Social and Environmental Health Research, London School of Hygiene and Tropical Medicine, London, WC1E 7HT UK

**Keywords:** Environmental determinants of health, Knowledge translation, Public health, Urban planning, Housing, Transport, Climate change, Air pollution, Integrated assessment

## Abstract

Cities around the world face many environmental health challenges including contamination of air, water and soil, traffic congestion and noise, and poor housing conditions exacerbated by unsustainable urban development and climate change. Integrated assessment of these risks offers opportunities for holistic, low carbon solutions in the urban environment that can bring multiple benefits for public health. The Healthy-Polis consortium aims to protect and promote urban health through multi-disciplinary, policy-relevant research on urban environmental health and sustainability. We are doing this by promoting improved methods of health risk assessment, facilitating international collaboration, contributing to the training of research scientists and students, and engaging with key stakeholders in government, local authorities, international organisations, industry and academia. A major focus of the consortium is to promote and support international research projects coordinated between two or more countries. The disciplinary areas represented in the consortium are many and varied, including environmental epidemiology, modelling and exposure assessment, system dynamics, health impact assessment, multi-criteria decision analysis, and other quantitative and qualitative approaches. This Healthy-Polis special issue presents a range of case studies and reviews that illustrate the need for a systems-based understanding of the urban environment.

## Background

Rapid urbanization, combined with rapid improvement in standards of living is stretching natural resources and threatening environmental quality in many countries. Population density has reached unprecedented levels in most parts of the high, medium and low income world. The urban population in 2014 was 54 % of the total global population, up from 30 % in 1950, and is projected to account for around 66 % of the global population by 2050 [1]. Urban areas are facing a range of environmental health challenges including contamination of air, water and soil. Sprawling urban areas contribute to traffic congestion, with associated air pollution, noise and long commuting times affecting public health and productivity across the world.

In addition, climate change is likely to aggravate certain urban health risks and inequalities by increasing the frequency and severity of extreme weather events (heatwaves, storms and floods), potentially contributing to air pollution episodes (ground-level ozone and pollen) and disturbing urban ecology [2, 3]. The urban heat island effect (i.e. the difference in temperatures between a city centre and the surrounding countryside) also exacerbates heat stress in built up areas [4]. This has knock-on effects on the indoor environment, energy demand (for ventilation and cooling) and public health [5, 6].

However, there is also an opportunity here: climate change mitigation and adaptation measures can deliver a range of health benefits. These health benefits are likely to result from “low carbon” policies aimed at lowering greenhouse gas emissions by improving energy efficiency in buildings (enhancing thermal comfort for occupants) [5], reducing dependency on private car use (improving physical activity levels and local air quality) [7], increasing renewable energy generation (improving ambient air quality) [8], and reducing meat and dairy consumption (reducing saturated fat intake) [9]. Accounting for the health co-benefits of climate change mitigation strengthens the case for reductions in greenhouse gas emissions from many sectors. However, attention should also be paid to the unintended harmful effects of certain carbon reduction policies. For example, home energy efficiency measures have the potential to worsen indoor air quality if steps are not taken to maintain good ventilation [10]; and the promotion of active travel has the potential to increase road injury risks without separation of cyclists and pedestrians from other road traffic [7].

Cities are complex systems. Research to elucidate pathways to better health and wellbeing demands systems-based, interdisciplinary methods involving epidemiologists, toxicologists, urban planners, environmental scientists, mathematical modellers, engineers, IT experts, social scientists, public health researchers and health care professionals. Importantly, local communities need to be involved in research projects aiming to inform local policies from an early stage. This can be achieved through genuine stakeholder engagement [11], citizen science and knowledge co-generation approaches [12], which raise awareness, provide valuable information and improve acceptability of interventions.

Methodological innovation in epidemiology, exposure assessment and risk analysis, and standardization of methods across countries, are needed to address complex environmental health challenges in the context of climate change and sustainable development. Relevant areas include the assessment and reduction of the health risks and impacts of weather extremes, air pollution, water contamination and other forms of environmental hazard, especially in the context of climate change, and evaluating mitigation and adaptation options [13]. These challenges highlight the need for integrated assessment methods that account for the complex interactions (including feedback loops) between climatic, environmental and behavioural factors, and the urban fabric [14]. This is particularly the case in global megacities where exposure to environmental stressors (such air pollution, congestion, heat and noise) can be substantially higher than in rural areas. Particular opportunities for influencing development pathways may arise in the multitude of rapidly developing cities in low and middle income countries. System dynamics approaches [15] and multi-criteria decision analysis methods [16] integrating quantitative and qualitative evidence can help characterise the likely overall impacts of policy options in urban environments.

This is the approach adopted by Healthy-Polis (www.healthy-polis.org), a new international consortium for urban environmental health and sustainability which aims to: (1) promote innovation and standardization in research methods (including exposure modelling, environmental epidemiology, risk analysis and integrated assessment methods), (2) facilitate international, multi-disciplinary research collaborations, (3) provide training and promote capacity building especially in rapidly urbanizing countries, and (4) evaluate and promote environmental interventions to improve public health in cities.

A particular emphasis of Healthy-Polis is on engendering and supporting a growing community of young researchers in the field of urban environmental health, climate change and sustainability, who will push the research agenda forward through global collaborations in the coming critical decades.

## Methods and case studies

**Fig. 1:**
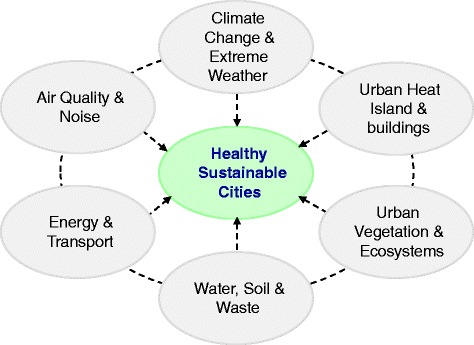
Healthy-Polis. Key areas of scientific research and inter-linkages in the urban environment

The disciplinary areas represented in Healthy-Polis are many and varied, including environmental epidemiology, modelling and exposure assessment, system dynamics, health impact assessment, multi-criteria decision analysis, and other quantitative and qualitative approaches. Key areas of interest (Fig. 1) were discussed at the 1^st^ Healthy-Polis workshop in Manchester, U.K. (6 March 2014).

In this special issue of Environmental Health, we present twelve contributions that address the aims of the Healthy-Polis consortium using methods from many disciplines. Perhaps the most familiar connection between climate change and health is the impact of extreme weather events such as heatwaves. A systematic review by Arbuthnott et al. [17] covers the important question of whether susceptibility to heat and cold has changed over time. It appears that various populations did become less susceptible to heat, although attribution to a specific cause is difficult. Heaviside et al. [18] consider the attribution of mortality to the Urban Heat Island effect during heatwaves, finding an appreciable contribution of this effect to the excess mortality experienced in the West Midlands region of England in the 2003 European heatwave. In regard to the co-benefits of climate change mitigation, Sabel et al. [19] report on the health benefits of several European and Chinese cities’ actual mitigation efforts, finding mixed results but with relatively modest health gains. The significant contribution of this study was in additionally considering climate change impacts on positive health outcomes, such as wellbeing.

We include a set of papers that address various aspects of disease in the urban environment. Asikainen et al. [20] focus on the calculation of the annual burden of disease caused by exposure to indoor air pollution in EU countries, and how best to ventilate with outdoor air, which may also be polluted. Considering various measures of urban form in 50 urban areas in England, Fecht et al. [21] intriguingly report higher rates of premature cardiovascular mortality in cities with higher densities of road junctions. Turning to infectious disease, Semenza et al. [22] present a predictive model of West Nile Virus infections based on ambient temperature and other environmental determinants. Higher rates are projected under climate change which has implications for the safety of the blood supply. Analysing the consequences of China’s massive ongoing migration and rapid urbanisation, Li et al. [23] show that action to protect and improve health in cities can be taken at multiple scales from national to individual.

Many of the Healthy-Polis papers address the broad area of urbanisation and planning. Macmillan et al. [24] report a project in which over 50 stakeholders collaboratively built causal diagrams to capture the complexities of housing, energy and wellbeing and developed criteria for assessing housing policy, while Nieuwenhuijsen [25] surveys new concepts and methods developed to address the complexity of urban environmental health in the wider context of urban and transport planning. Turning to specifics, Woods et al. [26] show how multi-criteria decision analysis can be used to prioritize environmental health hazards in a city. Salmond et al. [27] consider the ecosystem services and disservices provided by planting street trees as an urban planning tool, and argue that a holistic approach is necessary to ensure a net benefit. Finally, Rietveld et al. [28] argue for a systems approach to water and waste management in cities, illustrating their points with case studies from three continents.

## Conclusions and vision

The range of risks and opportunities for urban environmental health explored in this special issue clearly demonstrates the complexity of the challenge cities are facing in the 21^st^ century in the context of climate, land use and demographic change. As the planet becomes increasingly urbanised, pressure on natural resources (air, water, soil), urban infrastructure (housing and transport) and health care systems increases, but so does our capacity to address risks though technological innovation, international co-operation, and participatory decision-making at city level. Solutions may involve advanced “smart” systems (e.g. controlling energy consumption, temperature and ventilation in houses) as well as more traditional approaches (e.g. urban greening, promoting walking and cycling) to improve health and wellbeing. Importantly, these solutions need to be assessed in a holistic way to maximise the benefits (“win-win”, e.g. reducing energy consumption and improving thermal comfort and air quality in buildings) and avoid unintended trade-offs (“win-lose”, e.g. planting tree species that are aesthetically appealing but require high energy input for maintenance). Methods such as multi-criteria decision analysis, participatory system dynamics modelling and quantitative health impacts assessment can help avoid pitfalls of the past and create healthier and more sustainable cities. Healthy-Polis is committed to capitalizing on these opportunities by supporting international collaboration, building research capacity, and promoting dialogue between researchers, policy-makers and local communities.
